# Livin/BIRC7 expression as malignancy marker in adrenocortical tumors

**DOI:** 10.18632/oncotarget.14067

**Published:** 2016-12-21

**Authors:** Barbara Altieri, Silviu Sbiera, Silvia Della Casa, Isabel Weigand, Vanessa Wild, Sonja Steinhauer, Guido Fadda, Arkadius Kocot, Michaela Bekteshi, Egle M Mambretti, Andreas Rosenwald, Alfredo Pontecorvi, Martin Fassnacht, Cristina L Ronchi

**Affiliations:** ^1^ Department of Internal Medicine I, Division of Endocrinology and Diabetes, University Hospital of Wuerzburg, Germany; ^2^ Division of Endocrinology and Metabolic Diseases, Catholic University of the Sacred Heart, Rome, Italy; ^3^ Comprehensive Cancer Center Mainfranken, University of Wuerzburg, Germany; ^4^ Department of Pathology, University of Wuerzburg, Germany; ^5^ Division of Anatomic Pathology and Histology, Catholic University of the Sacred Heart, Rome, Italy; ^6^ Department of Urology, University Hospital of Wuerzburg, Germany; ^7^ Department of Anesthesiology and Critical Care, University Hospital of Wuerzburg, Germany

**Keywords:** livin, BIRC7, adrenocortical carcinoma, adrenal tumor, caspase-3

## Abstract

Livin/BIRC7 is a member of the inhibitors of apoptosis proteins family, which are involved in tumor development through the inhibition of caspases. Aim was to investigate the expression of livin and other members of its pathway in adrenocortical tumors and in the adrenocortical carcinoma (ACC) cell line NCI-H295R.

The mRNA expression of *livin*, its isoforms *α* and *β*, *XIAP*, *CASP3* and *DIABLO* was evaluated by qRT-PCR in 82 fresh-frozen adrenal tissues (34 ACC, 25 adenomas = ACA, 23 normal adrenal glands = NAG). Livin protein expression was assessed by immunohistochemistry in 270 paraffin-embedded tissues (192 ACC, 58 ACA, 20 NAG). Livin, CASP3 and cleaved caspase-3 were evaluated in NCI-H295R after induction of livin overexpression.

Relative *livin* mRNA expression was significantly higher in ACC than in ACA and NAG (0.060 ± 0.116 *vs* 0.004 ± 0.014 and 0.002 ± 0.009, respectively, *p* < 0.01), being consistently higher in tumors than in adjacent NAG and isoform *β* more expressed than *α*. No significant differences in *CASP3*, *XIAP* and *DIABLO* levels were found among these groups. In immunohistochemistry, livin was localized in both cytoplasm and nuclei. The ratio between cytoplasmic and nuclear staining was significantly higher in ACC (1.51 ± 0.66) than in ACA (0.80 ± 0.35) and NAG (0.88 ± 0.27; *p* < 0.0001). No significant correlations were observed between livin expression and histopathological parameters or clinical outcome. In NCI-H295R cells, the livin overexpression slightly reduced the activation of CASP3, but did not correlate with cell viability.

In conclusion, livin is specifically over-expressed in ACC, suggesting that it might be involved in adrenocortical tumorigenesis and represent a new molecular marker of malignancy.

## INTRODUCTION

Adrenocortical tumors (ACT) consist in frequent adenomas (ACA) and rare highly malignant carcinomas (ACC). ACC is one of the most aggressive solid tumors in humans, as evidenced by a 5-year survival rate of 25–50% in most series [1–3]. Effective treatment options for patients with advanced ACC are still scant. Thus, malignant adrenocortical tumors remain a major therapeutic challenge and new targets for treatment are urgently needed [4]. In addition, the histopathological diagnosis of ACC is usually based on multiple morphologic parameters, like the widely used Weiss diagnostic score [5], that are suggestive, but not always pathognomonic of malignancy [6]. Thus, although most adrenal tumors are not diagnostic dilemmas, there are still cases that are challenging [7] and novel molecular markers potentially leading to distinguish benign from malignant tumors are still needed.

The inhibitor of apoptosis proteins (IAP) family plays a key role in the regulation of apoptosis, cell proliferation and cell cycle control, mostly through the direct inhibition of specific caspases [8]. Currently, eight humans IAPs have been identified, including neuronal IAP (NAIP/BIRC1), cellular IAP1 (cIAP1/BIRC2), cellular IAP2 (cIAP2/BIRC3), X linked IAP (XIAP/BIRC4), survivin (BIRC5), BIR-containing ubiquitinin-conjugating enzyme (Bruce/Apollon/BIRC6), melanoma-IAP (ML-IAP/livin/BIRC7) and IAP-like protein 2 (ILP2/BIRC8). These proteins contain at least one of a highly conserved zinc-binding domain called BIR (baculovirus IAP repeat) motif, which is involved in protein-protein interaction and inhibition of caspases [9].

Livin/BIRC7 was first identified in melanoma [10] and consists of a single BIR domain that is homologous to the BIR3 domain of others IAPs [11], and a RING (Really Interesting New Gene) Zink finger domain. The gene *BIRC7* encodes two splicing variants: livin α and livin β, which are almost identical, except for 18 amino acids located in the BIR-RING interlinking region present only in the α isoform [12]. Despite this high similarity, the two isoforms are involved in the anti-apoptotic response to different stimuli. For example, livin α was associated with resistance against staurosporine while livin β was associated with resistance to etoposide, UV irradiation and TNF-α induced apoptosis [12, 13].

Livin exerts its anti-apoptotic effect especially through inhibition of caspase 3, but also of caspases 7 and 9 and of Smac/DIABLO (second mitochondria-derived activator of caspase/direct IAP binding protein with low p). Livin, binding DIABLO through its BIR domain, prevents also DIABLO–XIAP interaction, thus XIAP is also free to inhibit caspases. DIABLO is the major antagonist of IAP proteins [9], sensitizes tumor cells to apoptosis and controls the tumor growth and/or its metastatic spread [14]. This pro-apoptotic function has prompted the synthesis of Smac mimetics (peptides, polynucleotides and compounds) that target the BIR domain of IAPs and could be used in cancer therapy to either specifically induce apoptosis or act as drug-sensitizers [15, 16]. Such Smac mimetics, like the monovalent compounds GDC-0152 and GDC-0917 (CUDC-427) and the bivalent compound TL32711 (Birinapant) are being investigated in more than twenty phase I and II clinical trials in solid cancers and hematologic tumors (ClinicalTrials.gov).

Livin is highly expressed in fetal tissue and placenta [12] and in several tumor types, such as renal cell, bladder and colon cancer [17–19], hepatocellular carcinoma [20], non-small cell lung cancer [21], neuroblastoma [22] and childhood lymphoblastic leukemia [23]. Livin up-regulation is mainly a risk factor for cancer progression, poor prognosis and resistance to anti-tumor treatment [24, 25]. However, in some tumors, such as in childhood acute lymphoblastic leukemia, malignant mesothelioma, renal cell carcinoma and hepatocellular carcinoma, high livin expression is correlated with better prognosis [23, 26–28] or has no impact on clinical outcome [20, 29]. Furthermore, several studies demonstrated a negative correlation between the expression of livin and caspase-3 [24, 30, 31], which represents the main molecular target of livin action. Moreover, it has been shown that down-regulation of livin expression resensitizes tumor cells to apoptosis and chemotherapy [13, 25, 32] and leads to tumor volume reduction in a xenograft model of colorectal cancer [33]. For all these reasons, livin might represent a new potential target for future tumor-specific therapeutic strategy [9, 15, 34].

In a previous study on genomic alterations in adrenocortical tumors, we identified recurrent copy number gains at the region 20q13.3 (including the gene *BIRC7*) in 6/24 ACA (25%) and in 14/22 ACC (68%). Looking at other genes involved in the pathway of *BIRC7*, we found frequent copy number gains also in chromosomal region 12q (including *DIABLO*) in 11/22 ACC (50%) [35]. We previously demonstrated that survivin/BIRC5 has a higher expression in adrenocortical tumors than in normal adrenal glands and that its expression correlates with prognosis in ACC group [36]. Several studies indicated that livin and survivin play together an important role in the tumorigenesis and in the clinical outcome of several human cancers [18, 37]. The expression of other components of IAP family has been poorly evaluated in adrenocortical tissues [22].

The aim of the study was a comprehensive evaluation of livin/BIRC7 expression and its pathway in normal and neoplastic adrenal tissues, as well as in different adrenocortical cell lines, and its potential involvement in adrenocortical tumorigenesis. Moreover, we utilized the ACC reference cell line, NCI-H295R, to investigate *in vitro* the biological role of livin in the adrenocortical cell system.

## RESULTS

### *Livin*, isoforms *α* and *β, CASP3*, *DIABLO* and *XIAP* mRNA expression in adrenocortical tissues

**Figure 1:**
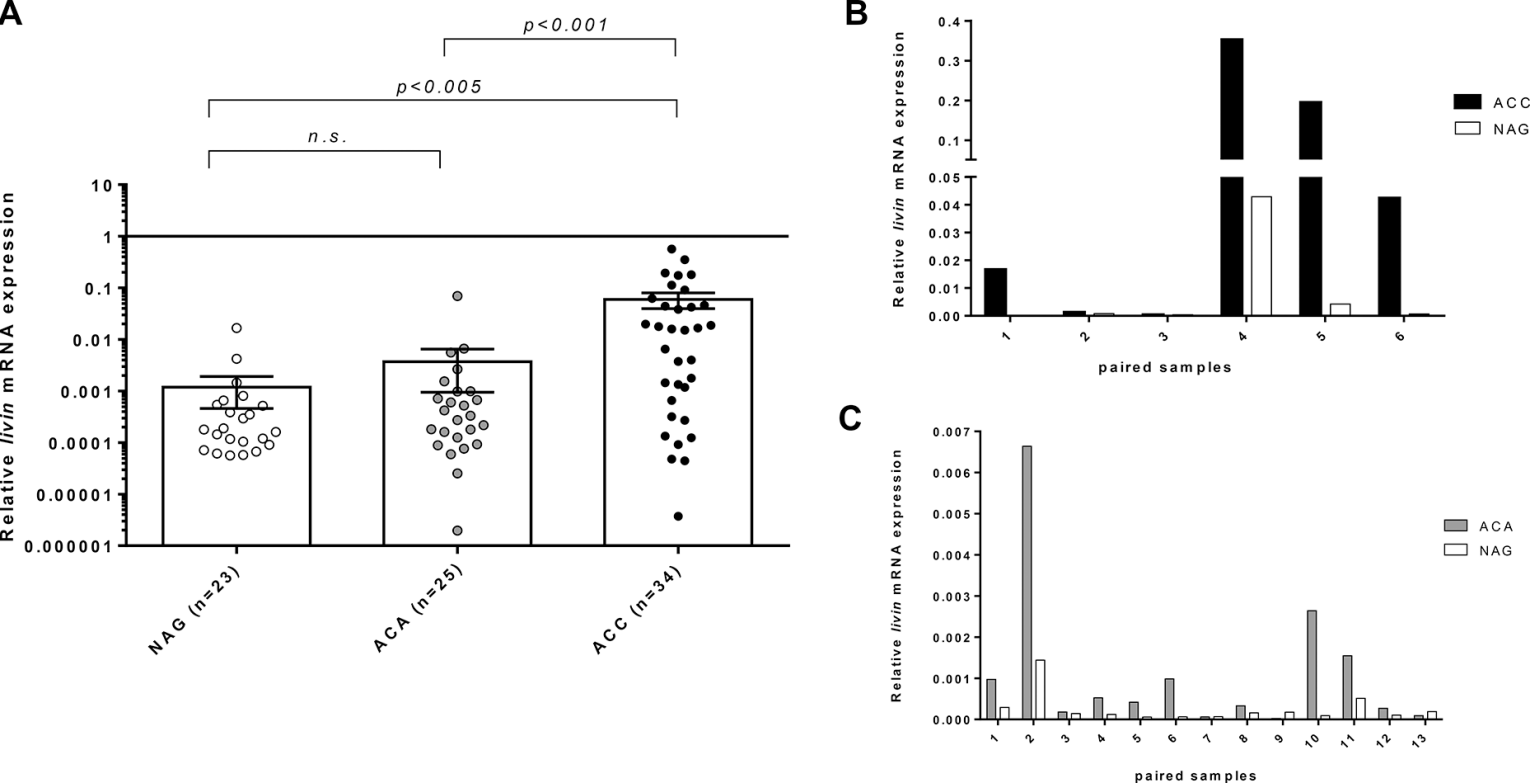
Relative *livin* mRNA expression levels by quantitative real time RT-PCR analysis Relative *livin* mRNA expression levels in normal adrenal glands (NAG), adrenocortical adenoma (ACA) and adrenocortical carcinoma (ACC) in the entire cohort of patients, expressed in log scale (**A**) and in 19 paired samples of tumor and corresponding NAG, of which 6 ACC (**B**) and 13 ACA (**C**) (*p < 0.005* per trend). Statistical analysis by Kruskall-Wallis test. N.s.= p not significant.

Relative *livin* mRNA expression was significantly higher in ACC (0.060 ± 0.116) than in both ACA and normal adrenal gland (NAG) (0.004 ± 0.014 and 0.002 ± 0.009, respectively, *p < 0.005*) (Figure 1A). There was no difference between NAG coming from adrenalectomies due to kidney cancer (*n* = 3, mean: 0.0002 ± 0.0001) and NAG adjacent to an adrenocortical tumor (*n* = 20, mean: 0.0026 ± 0.009, *p = n.s*.). Accordingly, when we analyzed the paired tumor/adjacent normal adrenal samples, *livin* was consistently higher in tumors (in both ACC and ACA) than in adjacent adrenal tissues (0.101 ± 0.131 *vs* 0.008 ± 0.016 and 0.001 ± 0.002 *vs* 0.0003 ± 0.0018 in ACC and ACA *vs* adjacent NAG, respectively; Figure 1B and 1C). Similar results were observed for *livin* isoforms *α* and *β*, which were both more expressed in ACC (0.019 ± 0.037 and 0.068 ± 0.121, respectively) than ACA (0.008 ± 0.036, *p = 0.005* for *livin α* and 0.020 ± 0.084, *p = 0.001* for livin *β*) and NAG (0.001 ± 0.004, *p < 0.001* for *livin α* and 0.004 ± 0.012, *p < 0.001* for livin *β*).

The relative *CASP3* mRNA levels were significantly higher in ACA (0.024 ± 0.012) than in ACC (0.017 ± 0.011, *p = 0.03*) and NAG (0.018 ± 0.012, *p = 0.05*). Instead, *XIAP* was significantly higher in NAG (0.033 ± 0.020) than ACC (0.022 ± 0.018, *p < 0.05*); no differences were found with ACA (0.028 ± 0.023). *DIABLO* mRNA levels were similar between NAG (0.245 ± 0.179) and adrenocortical tumors (0.241 ± 0.204 and 0.232 ± 0.189, respectively in ACC and ACA). For all these genes, we did not find any difference between paired samples of tumors and adjacent adrenal glands as we observed for *livin*.

Concerning the relationship with clinical parameters, we observed a significant inverse correlation between *CASP3* expression and tumor size (*p = 0.005*, R = 0.36). No other significant correlations were observed between the mRNA expression of investigated genes and clinical or pathological parameters including hormone secretion pattern, ENSAT tumor stage, Weiss score and Ki67 proliferation index. Moreover, no significant impact on progression-free and overall survival was found for all investigated genes (Supplementary Table S1).

### Livin α and livin β in adrenocortical tumors and adjacent normal adrenal glands

**Figure 2:**
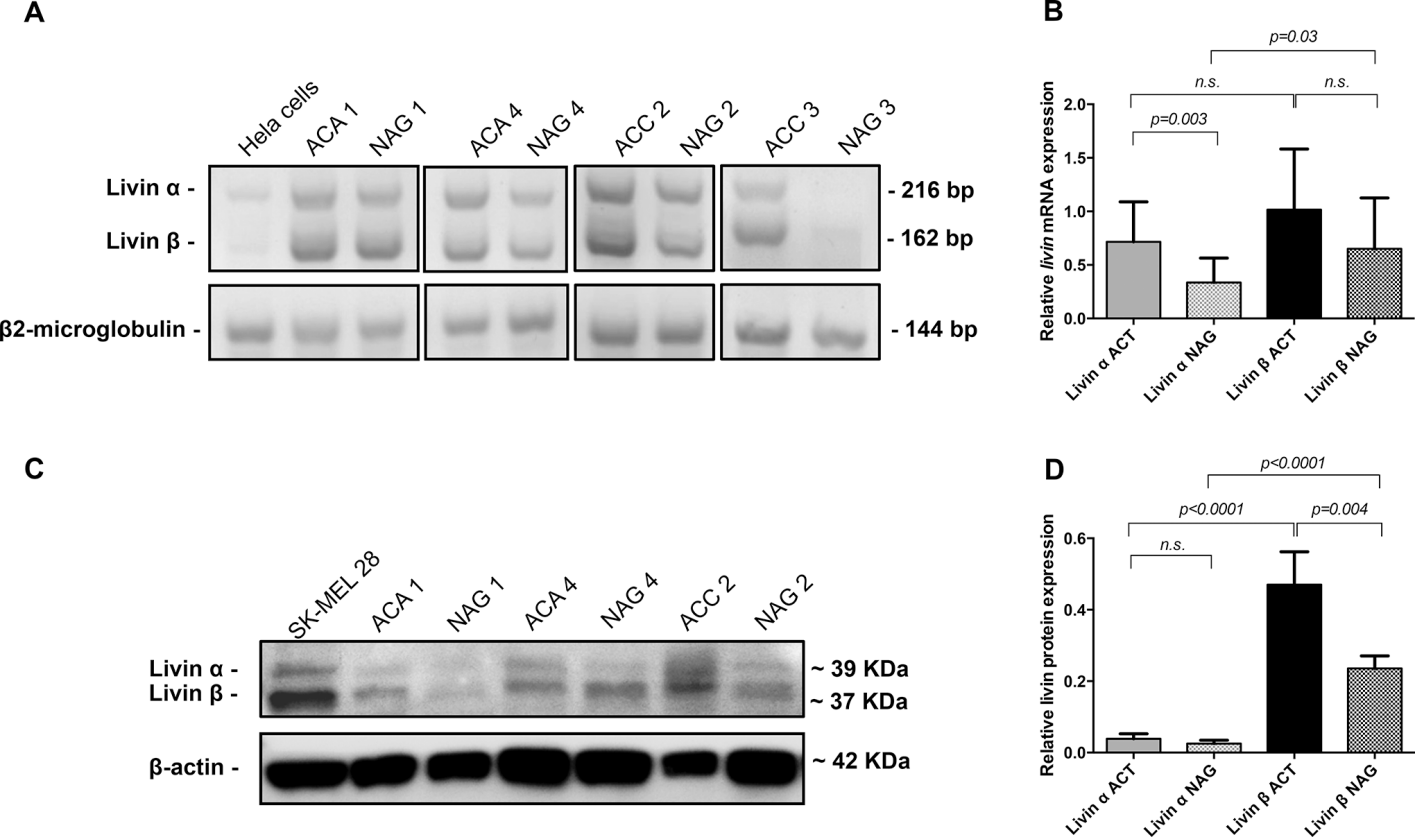
Expression of livin isoforms α and β in adrenocortical tumors and adjacent normal adrenal glands (**A**) 4× agarose gel for the expression of *livin* isoforms *α* and *β* at mRNA analyzed by RT-PCR in a subgroup of 4 paired samples of tumors (2 adrenocortical adenomas = ACA, and 2 adrenocortical carcinomas = ACC) and adjacent normal = NAG. Hela cells were used as positive control and *β2-microglobulin* was used as internal standard. (**B**) Quantitative analysis of agarose gel bands of all 15 paired samples of adrenocortical tumors (ACT) and corresponding NAG (*p = 0.3* per trend). Each bar in the histograms represents the mean of the ratio *livin α* or *livin β* signal to *β2-microglobulin* signal. (**C**) Western blot analysis of livin isoforms expression in three of four paired samples of tumors (2 ACA and 1 ACC) and adjacent NAG that are showed also at mRNA levels. Whole cell lysate SK-MEL 28 was used as positive control and β-actin was used as internal standard. (**D**) Quantitative analysis of WB bands of all 15 paired samples of tumors and corresponding NAG (*p < 0.0001* per trend). Each bar in the histograms represents the mean of the ratio livin α or livin β signal to β-actin signal. Statistical analysis by Kruskall-Wallis test and Mann-Whitney *U* test. N.s. = p not significant.

To better investigate the expression of livin isoforms in adrenal tissues, we compared livin α and livin β expression at mRNA level by RT-PCR (size differentiation agarose gel electrophoresis) (Figure 2A–2B) and protein level by Western Blot (WB) analysis (Figure 2C–2D) in 15 paired samples of adrenocortical tumors (10 ACA and 5 ACC) and adjacent adrenal glands. Both livin α and β were detected in all the 15 normal and neoplastic adrenocortical tissues with isoform β having generally a higher expression than the isoform α (Figure 2A and 2C).

At mRNA level, isoform *α* was significantly higher in tumors than in corresponding NAG (*p = 0.003*), while isoform *β* showed only a trend (*p = 0.07*) (Figure 2B). At protein level, livin β was significantly higher in tumors than in corresponding NAG (*p = 0.004*) and was significant higher than isoform α in both tumors (*p < 0.0001*) and NAG (*p < 0.0001*) (Figure 2D). No difference was observed for livin α protein expression between ACT and adjacent NAG (Figure 2D).

### Livin α and livin β in adrenocortical cancer cell lines

The relative *livin* mRNA expression was relatively low in all the ACC cell lines NCI-H295, NCI-H295R and SW13 compared to positive controls (Supplementary Figure S1A). The isoform *β* was higher expressed than the isoform *α* (Supplementary Figure S1B), similarly to what we reported for the adrenocortical tumors. The WB analysis showed moderate, but relatively low, livin protein expression levels in NCI-H295R cells (Supplementary Figure S1C).

### Immunohistochemical evaluation of livin protein expression

**Figure 3:**
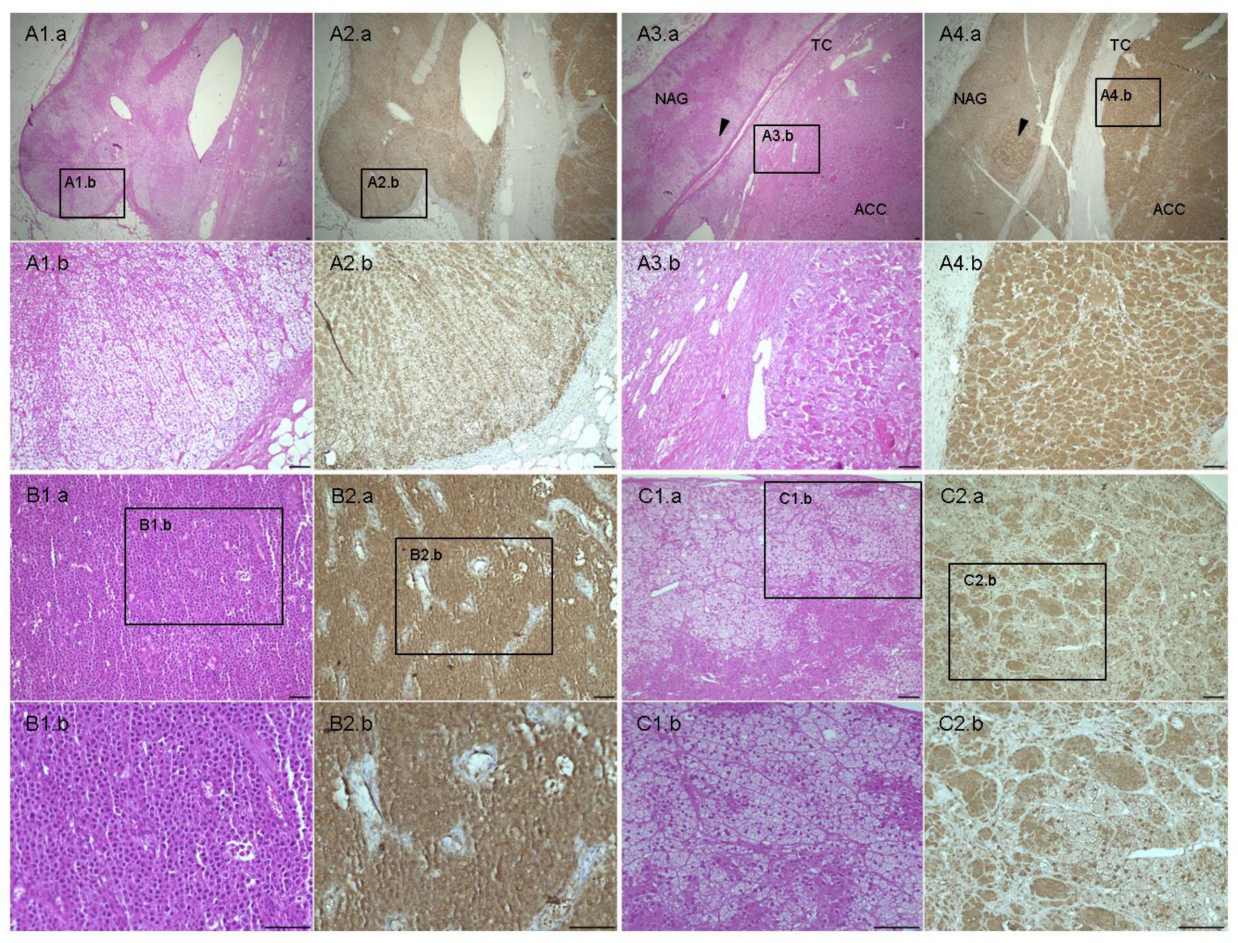
Hematoxylin & eosin and livin staining in adrenal samples (**A**) Normal adrenal gland (NAG) (A.1–2) with adjacent adrenocortical carcinoma (ACC) (A 3-4) stained for H&E (A.1 and A.3) and livin (A.2 and A.4). A.1b, 2b and A.3b, 4b are 10× enlarged and detailed images of the 2× NAG and ACC, respectively. Arrows point to tumor infiltration in the adjacent NAG. TC: capsule, separating NAG and ACC. (**B**) Example of ACC with H-score 3 and negative nuclei stained for H&E (B.1) and livin (B.2) in comparison with (**C**) adrenocortical adenoma (ACA) characterized by H-score 2 and positive nuclei stained for H&E (C.1) and livin (C.2). The .1b, 2b and C.1b, C.1c are 20× enlarged and detailed images of the 10× ACC and ACA, respectively. Scale bar: 100 μm.

**Figure 4:**
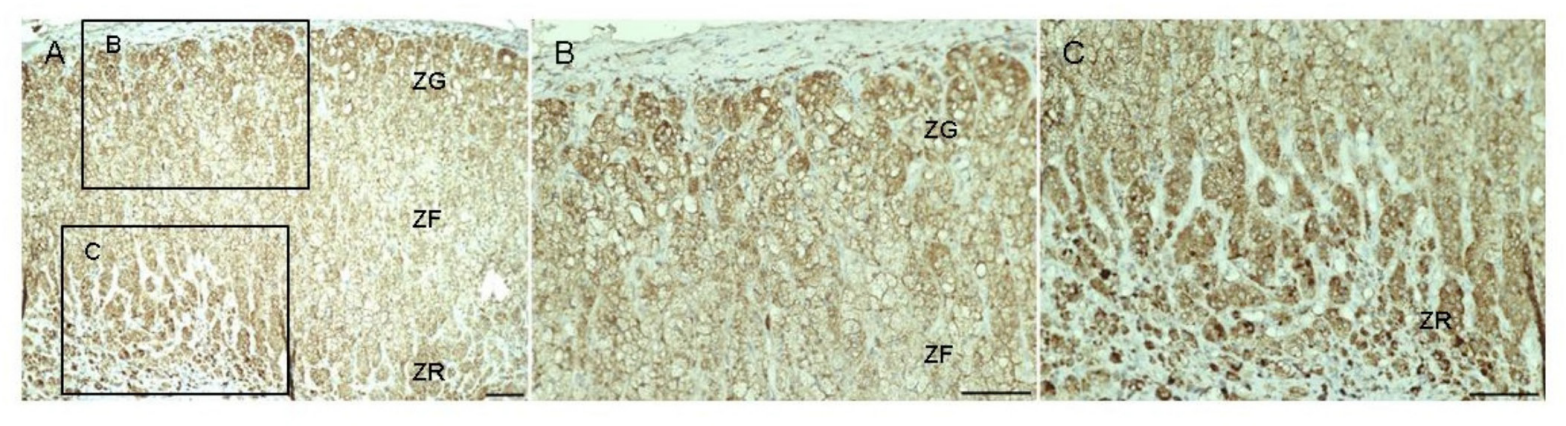
Livin staining in normal adrenal gland Livin staining in normal adrenal gland (**A**). It is possible to recognize the three different zone of the adrenal gland: the zona glomerulosa (ZG), the zona fasciculata (ZF) and the zona reticularis (ZR). The Figures (**B**) and (**C**) are 20× enlarged and detailed images of the 10× ZG, ZF and ZR, respectively. Scale bar: 100 μm.

As expected, livin cytoplasmic staining was higher in tumors than in adjacent normal tissue in all studied samples, including skin (Supplementary Figure S2A), kidney (Supplementary Figure S2B), adrenal gland (Figure 3A1–3A4), colon and testis. Renal cell carcinoma and melanoma lymph node metastasis showed the strongest staining intensity (H-score 3). Livin staining was detected also in some specific cells of normal tissues, such as glomerular mesangial cells, podocytes and tubule epithelial cells in normal kidney (Supplementary Figure S2B. S2a–b), crypt epithelial cells in normal colon, gastric glands in normal stomach and seminiferous tubules in normal testis. Similarly, we observed a positive livin immunostaining also in hormone-secreting cells of the cortex and the medulla of the normal adrenal gland, but not in the stroma and in the capsular tissue (Figure 3A1–2 and Figure 4). Specifically, the zona glomerulosa and fasciculata (Figure 4A1–2) showed a cytoplasmic staining (both mean H-score: 1.60 ± 0.89) and positive nuclei in less than 50% of cells (mean nuclear score: 0.80 ± 0.84 and 1.20 ± 0.84, respectively), while the zona reticularis showed the highest staining in both cytoplasm (mean H-score: 2.20 ± 0.84) and nuclei (mean nuclear score: 1.60 ± 0.55) (Figure 4A3). The medulla presented higher immunostaining than adrenal cortex, both at cytoplasm (mean H-score: 2.70 ± 0.58) and nuclear level (mean nuclear score: 1.60 ± 0.55).

**Figure 5:**
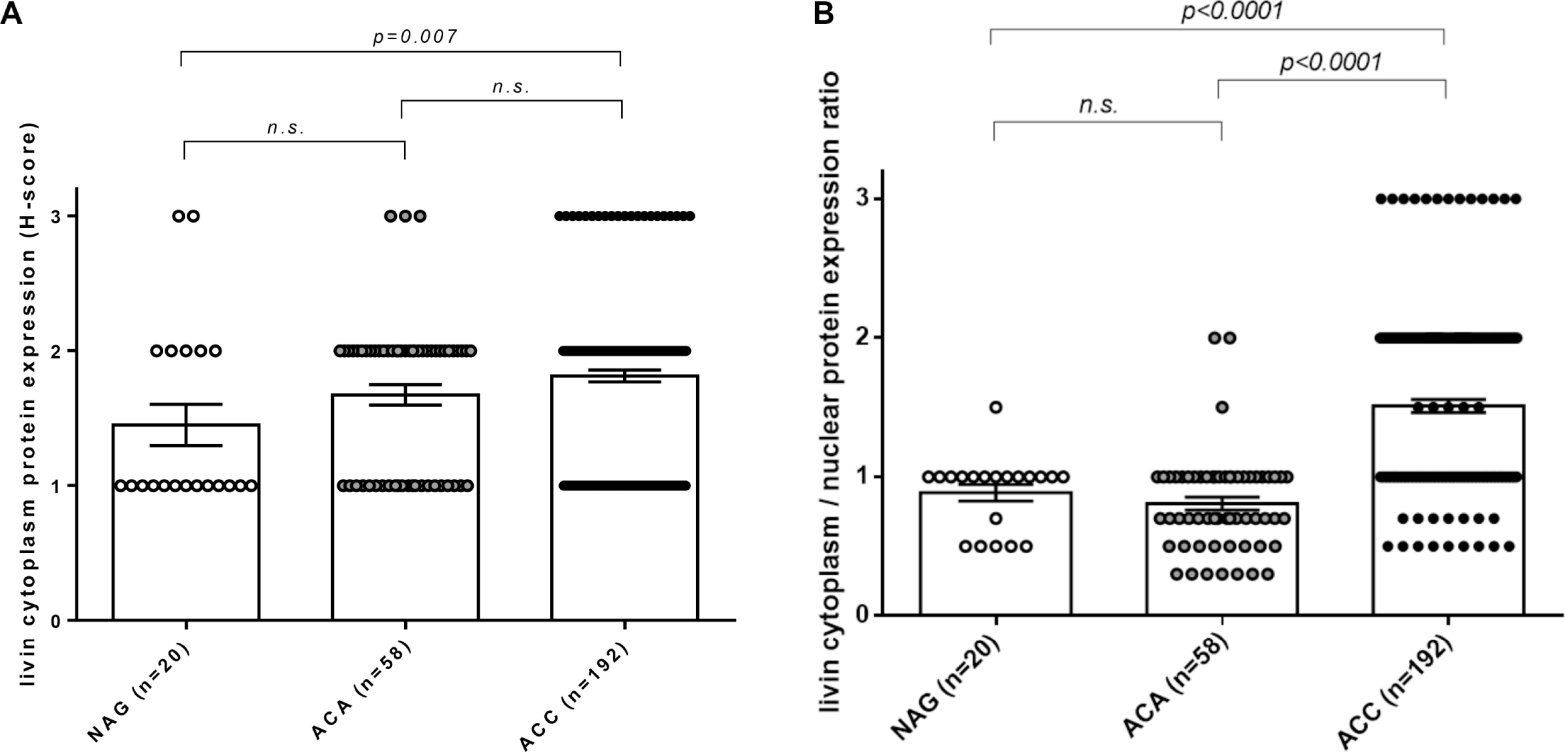
Immunohistochemistry of cytoplasmic livin expression and cytoplasmatic/nuclear ratio in adrenocortical tissues (**A**) Livin cytoplasmic protein expression evaluated as H-score in normal adrenal glands (NAG, *n* = 20), adrenocortical adenomas (ACA, *n* = 58) and adrenocortical carcinomas (ACC, *n* = 192) (*p = 0.01* per trend). (**B**) Livin cytoplasm/nuclear protein expression ratio evaluated as the ratio between H-score and nuclear score in NAG (*n* = 20), ACA (*n* = 58) and ACC (*n* = 192) (*p < 0.0001* per trend). Statistical analysis by Kruskall-Wallis test. N.s. = p not significant.

Moreover, livin cytoplasmic immunostaining was significantly higher in ACC (mean H-score: 2.00 ± 0.61) than in both ACA (1.67 ± 0.57) and NAG (1.45 ± 0.69, *p = 0.01* per trend by Kruskall-Wallis test) (Figure 5A). However, no significant difference was found between the ACC and ACA group. In the ACC group, no difference in livin expression was observed among primary tumors, local recurrences and metastatic tissues (data not shown). Contrary to the cytoplasmic staining, livin nuclear staining was clearly higher in ACA (Figure 3C1–2) (mean nuclear score: 2.28 ± 0.72) than in ACC (1.34 ± 0.57) (Figure 3B1–2), and NAG (1.70 ± 0.66) (*p < 0.0001* per trend by Kruskall-Wallis test). Interestingly, we observed that the ratio between cytoplasmic and nuclear staining was significantly higher in ACC (mean ratio: 1.51 ± 0.66) than in ACA (mean ratio: 0.80 ± 0.35) and NAG (mean ratio: 0.88 ± 0.27) (*p < 0.0001* per trend by Kruskall-Wallis test), while no significant difference was found between ACA and NAG (Figure 5B).

We did not observe any significant correlations between both cytoplasmic and cytoplasmic/nuclear ratio protein level and clinical or pathological parameters, including hormone secretion pattern, ENSAT tumor stage, Weiss score, Ki67 proliferation index, and number of distant metastasis. No correlations were observed between livin isoforms *α* and *β* at mRNA level and cytoplasmic or nuclear staining. No impact on clinical outcome was found for both cytoplasmic and cytoplasmic/nuclear ratio staining (Supplementary Figure S3A–S3D).

In a total of 31 adrenocortical tumors (10 ACA and 21 ACC) we evaluated the livin expression at both mRNA and protein level, observing a strong positive correlation (Supplementary Figure S4). In particular, cytoplasmic livin protein staining was higher in ACT showing high *livin* mRNA levels (*n* = 16, of which 14 with H-score 2–3) as compared with ACT with low mRNA levels (*n* = 15, of which 9 with H-score 2–3, *p < 0.0001* by Chi-squared test).

### Relationship between livin cytoplasmic staining and copy number gains

A total of 32 adrenocortical tumors (17 ACA and 15 ACC) were evaluated in a previous study by single nucleotide polymorphism (SNP) array analysis profiling [35]. In this group, we observed a recurrent copy number (CN) gain at the region 20q13.3 including the gene *BIRC7* in 6 ACA and 8 ACC. Overlapping the expression data on the CN data, we observed a trend to a positive correlation between CN status and cytoplasmic livin protein expression. Specifically, the staining was higher, even if not significantly, in ACT affected by CN gain (*n* = 14, of which 12 with H-score 2–3) as compared with ACT with normal CN status (*n* = 18, of which 11 with H-score 2–3, *p = 0.234* by Chi-squared test with absolute values).

### Relationship between cytoplasmic localization of livin and survivin stainings

In a subgroup of 146 adrenal samples, we observed a strong relationship between the cytoplasmic livin and survivin staining (*p = 0.0123* calculated by Chi-squared test) (Supplementary Figure S5). Specifically, survivin expression was low (H-score 0–1) in 31 tissue samples (including 24 ACC, 4 ACA and 3 NAG), of which 14 samples also had low cytoplasmic livin, whereas survivin was high (H-score 2–3) in 115 tissue samples (comprising 94 ACC, 16 ACA and 5 NAG), of which 95 cases had also high livin cytoplasmic.

In a subgroup of 93 ACC cases deriving from primary tumors, we evaluated the impact of the two proteins on clinical outcome. However, we did not observe any significant impact on overall and progression free survival by combining the expression of the two proteins (data not shown).

### Effect of livin overexpression on adrenocortical cells *in vitro*

**Figure 6:**
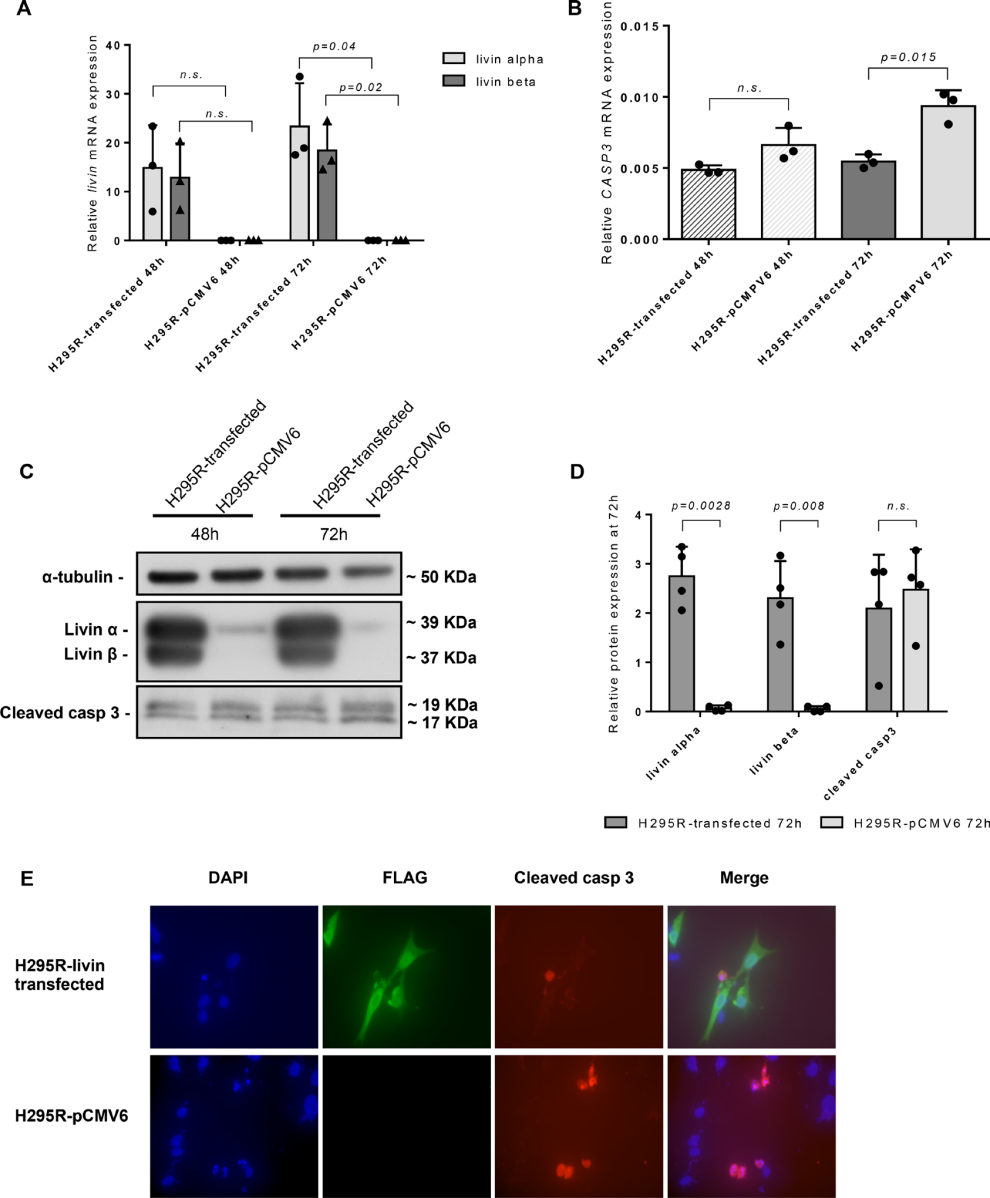
Effect of livin overexpression on adrenocortical cell line NCI-H295R *in vitro* Relative *livin* mRNA levels (**A**) and *CASP3* mRNA levels (**B**) evaluated by qRT-PCR at both 48 h and 72 h in NCI-H295R cells after livin transfection in comparison with those transfected with the empty vector (pCMV6). All qRT-PCR experiments were conducted in triplicates. (A) After 72 hours from transfection, the mRNA level of *livin α* and *livin β* were significantly increased in *livin*-NCI-H295R cells in comparison with those transfected with empty vector (*p = 0.04* and *p = 0.02* for *livin α* and *livin β*, respectively). (B) Livin overexpression was associated with a significant decrease of *CASP3* mRNA levels after 72 hours from transfection (*p = 0.015*). (**C**) WB analysis showed clearly higher livin α and β protein levels and a light decrease of cleaved caspase 3 protein levels at both 48 and 72 hours. (**D**) Quantitative analysis of livin α, livin β and cleaved caspase-3 by WB in *livin*-NCI- H295R transfected cells in comparison with those transfected with the empty vector after 72 h. Livin α and livin β were significantly increased in *livin*-NCI-H295R cells in comparison with pCMV6-NCI-H295R (*p = 0.0028* and *p = 0.008* for livin α and livin β, respectively). Cleaved caspase-3 was only slightly decreased in *livin*-NCI- H295R transfected cells (*p = n.s*.). Each bar in the histograms represents the mean of the ratio between cleaved casp3 signal and α-tubulin signal. Each point represents the value of this ratio deriving from four separate experiments. (**E**) Immunofluorescence staining showed a decrease of cleaved caspase-3 protein levels (red color) in *livin*-NCI-H295R cells (green color) compared to pCMV6-NCI-H295R. Cell nuclei were visualized with 4#-6-diamidino-2-phenylindole (DAPI, blue color). Magnification 60×. Statistical analysis by unpaired *t-test* with Welch's correction. n.s.= p not significant.

Livin overexpression was successfully induced in NCI-H295R cells (*livin*-NCI-H295R) (Figure 6A, 6C and 6E). Accordingly, after 48 and 72 hours from transfection, the *livin α* and *livin β* mRNA and protein levels were increased in *livin*-NCI-H295R cells in respect to those transfected with the empty vector (pCMV6-NCI-H295R; Figure 6A and 6C). Particularly, after 72 hours from transfection, livin isoforms were significantly increased at both mRNA (23.32 ± 8.86 *vs* 0.000013 ± 0.000005, *p = 0.04* and 18.42 ± 5.28 *vs* 0.000070 ± 0.000042, *p = 0.02* for *livin α* and *livin β*, respectively, by unpaired *t-test* with Welch's correction, Figure 6A) and protein levels (2.75 ± 0.59 *vs* 0.06 ± 0.06, *p = 0.028* and 2.30 ± 0.75 *vs* 0.01 ± 0.01, *p = 0.008* for *livin α* and *livin β*, respectively, by unpaired *t-test* with Welch's correction, Figure 6C–6D) in *livin*-NCI-H295R cells in comparison to control pCMV6-NCI-H295R cells.

To determine the impact of livin overexpression on apoptosis, we evaluated *CASP3* expression at mRNA level by qRT-PCR and cleaved caspase-3, the active form of this enzyme, at protein level by both WB and immunofluorescence (IF). We observed that *CASP3* mRNA levels significantly decrease in *livin*-NCI-H295R in comparison with pCMV6-NCI-H295R cells after 72 hours from transfection (0.0055 ± 0.0005 *vs* 0.0093 ± 0.0011, *p = 0.015* by unpaired *t-test* with Welch's correction; Figure 6B). At WB analysis (Figure 6C–6D), the cleaved caspase-3 was partially decreased in *livin*-NCI-H295R in comparison to pCMV6-NCI-H295R cells both at 48 and 72 hours after transfection (2.09 ± 1.09 *vs* 2.48 ± 0.81, *p = n.s*. at 72 hours by unpaired *t-test* with Welch's correction; Figure 6D). IF staining after 72 hours from transfection, revealed in the evaluated cells a significant reduction of cleaved caspase-3 in the *livin*-NCI-H295R cells (mean percent of cleaved caspase-3 positive cells: 10.76 ± 6.41 *vs* 23.84 ± 11.51, *p = 0.003* for *livin*-NCI-H295R and pCMV6-NCI-H295R cells, respectively, by unpaired *t-test* with Welch's correction; Figure 6E).

A cell viability test was performed at 48 and 72 hours after transfection to assess the direct potential role of livin on cell proliferation. Importantly and according with the results of the immunostaining in tissue samples, we did not find any significant difference between the *livin*-NCI-H295R and pCMV6-NCI-H295R (data not shown).

## DISCUSSION

Livin/ML-IAP/BIRC7 is a member of the inhibitor of apoptosis proteins family, which plays a key role in the regulation of apoptosis and modulation of cell cycle and cell proliferation. Livin is over-expressed in several cancer types and presents an anti-apoptotic activity mediated mostly by the direct inhibition of caspase 3, but also of caspases 7 and 9 and DIABLO. To our knowledge, this is the first study that systematically investigates the expression of livin/BIRC7 and different members of its pathway in normal and neoplastic adrenocortical tumors. In particular, we could demonstrate that livin is more expressed in ACC than in ACA and NAG, both at mRNA and protein level. These results are conform to gene expression data extracted from the high density oligonucleotide array of adrenocortical tumors published by Giordano and colleagues [38] and deposited in National Center for Biotechnology Information´s gene Expression Omnibus (accession number GSE10927) which showed that *livin* is significantly higher expressed in ACC in comparison to ACA and normal adrenal glands (Supplementary Figure S6). Similarly, in a very recent multicentric study regarding a comprehensive genomic characterization of a large series of 80 ACC [39], the authors found a relatively high *livin* mRNA expression by RNA-sequencing (median log fold change = 5.96). All these findings suggest a potential major role of livin in the adrenocortical tumor development, as previously reported for other cancer types [18–20, 29, 40–42].

Interestingly, we also observed a significant positive correlation between the *livin* mRNA expression and protein levels as evaluated by immunohistochemistry, in contrast to what was previously reported by Lazar et al. in melanoma [43]. As demonstrated in renal cell carcinoma, in hepatocellular carcinoma and colorectal cancer [19, 20, 40], we were able to show that livin is upregulated in tumor tissues compared to the adjacent normal adrenal gland, again both at mRNA and protein level. In contrast to Kim and colleagues [22], we detected livin staining in hormone-secreting cells of normal adrenal glands, not only in medulla but also in the adrenal cortex.

BIRC7 gene encodes two splicing variants, livin α and livin β, known to have different anti-apoptotic properties *in vitro* [12, 13] and also a different expression in tissues. Particularly, livin β seems to be more effective than the α isoform in blocking apoptosis induced by DNA damaging agents such as etoposide [12]. We observed that adrenocortical tumors and normal adrenal glands expressed both livin α and β isoforms, at both mRNA and protein level. This is in agreement with studies performed in renal cell carcinoma and in hepatocellular carcinoma [20, 40] and in contrast to bladder cancer, where only the α subunit is expressed [18]. Furthermore, we demonstrated that livin β is more expressed than the α isoform in both adrenocortical tumors and normal adrenal glands. However, we did not observe any significant correlation between the two isoforms and the clinical and pathological features. Further studies are needed to better elucidate the pathological role of the two livin isoforms in adrenocortical tumors.

We did not found any significant correlation between livin expression at both mRNA and protein levels, and the histopathological and/or clinical parameters, including the clinical outcome as observed in some [22, 27, 42], but not all other cancer types [20, 29, 44, 45]. Our results are in accordance to other recently published gene expression data in ACCs [39], where no significant relationship was observed between livin expression and overall survival (*p = 0.86*). This discrepancy compared to other human cancers may be at least partially due to the diverse antibodies and scoring system used in the previous studies, mostly including only small series of patients. In the present study, we used a highly specific antibody against the full-length recombinant human livin well suitable for immunohistochemistry and investigated a very large series of normal and neoplastic adrenocortical tissues. Furthermore, a major point to be considered is that livin does not regulate the apoptosis process alone, but it is part of a complex apparatus where several physio-pathological mechanisms are involved. Hence, *in vitro* experiment in the livin transfected ACC cell line NCI-H295R confirmed that livin overexpression was not able to induce cell proliferation or modifications in cell viability. This is also reflected by the fact that livin expression also did not correlate with survival in adrenocortical cancer. Additionally, in H295R cells, the livin overexpression induced a down-regulation of the expression of *CASP3* at mRNA level. At the protein level, we evaluated the expression of cleaved caspase-3, which is a major effector of the intrinsic and extrinsic pathway of apoptosis, by IF analysis and WB. Although we observed in IF a significant reduction of the active caspase-3 in the evaluated cells, we did not obtain a significant decrease of cleaved caspase-3 at WB analysis. This difference could be related to the different sensitivity of the two methods, but we cannot exclude that livin overexpression produces only a slight decrease of the active form of caspase-3. Thus, we hypothesize that livin may be able to modulate apoptosis mainly via the partial inhibition of caspase-3 also in adrenocortical cells, as previously reported in other cancer types [24, 30, 31]. So while livin does not lead to an increased malignity of the adrenocortical tumors, it might affect the cellular resistance to apoptosis, which may play an important role during treatment. However, not surprisingly, also additional mechanisms are likely to be involved in the complex mechanism of the apoptotic cascade in ACC.

An open question is represented by the different distribution of the livin immunostaining between benign (preponderant positive nuclei) and malignant adrenocortical tumors (preponderant positive cytoplasm). As showed for other cancer types [19, 27, 40, 41, 45], we observed livin localized in both cytoplasm and nuclei. However, none of the previous observations described a significant difference in livin distribution between adenomas and carcinomas. Moreover, we report also a different distribution of livin staining among the three cortical zones and the medulla of the normal adrenal gland. The biological significance of these observations remains unclear [46]. To our knowledge, this is the first study which describes a difference in livin staining among normal tissue, benign and malignant tumors in a large series of adrenal tissues. As described in literature, livin is a regulator rather than an inhibitor of apoptosis and it could have both antiapoptotic or proapoptotic activity in the same tumor type based on protein levels [43] or cellular localization [46]. Nachmias and colleagues demonstrated that the full-length livin protein, which is associated with anti-apoptotic activity, is detected exclusively in the cytoplasm, whereas the accumulation of truncated livin in the nucleus is correlated with an increase in apoptosis [46]. The antibody we used for immunohistochemistry staining recognizes both the full-length and the truncated form of the livin protein. Thus, we are not able to distinguish between the anti-apoptotic, that could be associated with malignant tumors, and the pro-apoptotic livin form, which could be associated with benign tumors. However, we demonstrate that the ratio between cytoplasmic and nuclear staining was significantly higher in ACC than in ACA and NAG, while no significant difference was found between ACA and NAG. Here we may speculate that this different cell distribution of livin could be related with its pleiotropic activity in the regulation of the complex pathway of apoptosis that needs to be further investigated. Nevertheless, a different localization/distribution of livin inside the cells might be useful for the differential diagnosis between benign and malignant adrenocortical tumors, particularly when the lesion tends to be difficult to classify with classical histopathological methods, mostly based on Weiss score.

In terms of pathogenetic mechanisms, we observed a trend to a positive correlation between the livin protein levels and the *BIRC7* copy number status. Specifically, the adrenocortical tumors with copy number gains in the chromosomal region including the *BIRC7* gene showed a higher livin expression, thus suggesting a potential causative genomic alteration in this segment.

We also observed a significant positive correlation between the livin and the survivin protein expression levels. The two IAPs are commonly overexpressed in several malignant tumors and both modulate apoptosis through the inhibition of caspases. Many studies indicated that survivin and livin play together an important role in tumorigenesis and are correlated with clinical outcome in human cancer [18, 47, 48]. However, in our series, only the survivin expression showed a significant negative impact on overall survival in patients with ACC, similarly to what was reported in previous studies in other cancer types [44, 45].

The high livin expression levels in malignant adrenocortical tumors also suggests that it might represent a novel potential drug target for patients with ACC [9, 49, 50]. Because IAP members block apoptosis at the down-stream effector phase, a point where multiple signaling pathways converge, they represent attractive molecular targets for the design of new classes of anticancer drugs aimed to overcome apoptosis resistance of cancer cells [8, 16]. Several strategies to target livin in novel cancer therapies have been investigated, such as antisense nucleotides [51] and cancer immunotherapy [52]. However, it is worth noting that targeting livin might cause different side effects due to its known expression in several normal tissues (i.e. nephrotoxicity, gastrointestinal disorders, infertility, or adrenal disorder) [20, 22, 29, 40, 41]. While not strictly tumor-specific, the overexpression of livin in tumor cells represents a preferential rather than a specific cancer target that needs to be further investigated. This topic will be the object of future research projects.

In conclusion, our findings demonstrate that livin is specifically over-expressed in ACC, suggesting that it might be involved in adrenocortical tumorigenesis and it might represent a possible new molecular marker of malignancy in adrenocortical tumors. Moreover, as previously reported for different human cancers, these findings might open a new perspective for the use of livin as a potential therapeutic target in ACC that deserves to be further investigated.

## MATERIALS AND METHODS

### Tissue samples and clinical characteristic

A total of 82 fresh-frozen adrenal tissues, collected between October 2006 and October 2014, were used for the evaluation of *livin*, its isoforms α and β, *CASP3, XIAP* and *DIABLO* mRNA levels. In particular, 23 (NAG) deriving from the area surrounding the tumors (*n* = 20) or from adrenalectomies performed during surgery for renal carcinoma (*n* = 3) and 59 adrenocortical tumors (25 ACA and 34 ACC) have been investigated. Among these, 19 were paired samples of tumors and corresponding adjacent normal adrenal glands (13 ACA and 6 ACC). In a subgroup of 15 out of 19 paired samples with enough material (10 ACA and 5 ACC), livin protein expression was also investigated by WB analysis. Among the ACC group, 29 tissues were primary tumors, 1 local recurrence and 4 distant metastases. The last follow-up was January 2016. Patients undergone adrenalectomy for primary tumor presented a median follow-up of 31 months (range 3–189 months). Among these, 13 were died for the disease, 15 were still alive at the last follow-up and 1 was lost from the follow-up.

For the livin immunohistochemistry analysis, we investigated 314 paraffin-embedded tissue sections (including 192 ACC, 58 ACA, 20 NAG, 6 other normal tissues, 38 other cancers), comprising 171 standard full slides and 143 assembled in three tissue microarrays (TMA). TMA were assembled as previously reported [53] and only patients with at least 2 out of 5 evaluable cores in the TMA after the staining procedure were included in the final series. A total of 250 adrenocortical tumor samples were evaluated. These adrenocortical tumor tissues included 32 samples (17 ACA and 15 ACC) that had been investigated in a previous SNP array analysis [35] and which were used for the comparison between copy number alteration and protein expression. Among the ACC group, 147 tissues were primary tumors, 25 local recurrences and 20 distant metastases. The last follow-up was January 2016. Patient underwent to adrenalectomy for primary tumor presented a median follow-up of 37 months (range 1–224 months). Among these, 86 were died for the disease, 57 were still alive at the last follow-up and 4 were lost from the follow-up.

For the comparison between livin protein staining and mRNA levels, we evaluated a total of 31 tissue samples, including 10 ACA and 21 ACC. We also investigated other non-adrenal tissues, comprising 6 normal tissues (liver, ovary, uterus, stomach and 2 samples of tonsils) and 38 tissues from several cancers (melanoma, lymphoma, renal cell carcinoma, bladder cancer, colon cancer, hepatocellular cancer, pancreatic cancer, breast cancer, ovarian cancer, testicular cancer, prostatic cancer, bronchial cancer and non-small cell lung cancer) as controls.

Clinical parameters, such as sex, age at diagnosis, tumor size, hormone secretion pattern, pathological classification and, in case of ACC, tumor stage according to the European Network for the Study of Adrenal Tumors (ENSAT) classification [54], Weiss score, Ki67 proliferation index, presence and number of distant metastasis, clinical outcome were collected through the German ACC and the ENSAT Registry (www.ensat.org). Hormonal hypersecretion and malignancy of the tumors were defined according to established clinical, biochemical and pathological criteria [5, 55]. Clinical parameters and tumor characteristics are summarized in Table 1.

**Table 1:** Clinical and pathological characteristics of both cohorts of patients for (A) mRNA and (B) protein analysis

A. Cohort of patients for mRNA analysis by qRT-PCR						
	ACA		ACC		*p* value	
*n*	25		34		*n.s*.	
Sex (F:M)	16:9		18:16		*n.s*.	
Age yrs-median (range)	49.5 (25–69)		50 (22–80)		*n.s*.	
Tumor size cm-median (range)	3.2 (0.9–6.5)		7.6 (2.5–24.0)		*< 0.05*	
Hormone secretion - *n* available	25		34		*n.s*.	
Cortisol – *n* (%)	11 (44%)		15 (44%)			
Androgen – *n* (%)	0 (0%)		4 (12%)			
Aldosterone – *n* (%)	7 (28%)		1 (3%)			
Inactive – *n* (%)	7 (28%)		14 (41%)			
Tumor localization	–				n.a.	
Primary tumor			29			
Local recurrences Metastases			1 4			
ENSAT Tumor Stage-*n* available	–		32		n.a.	
I–II – *n* (%)			13 (41%)			
III – *n* (%)			11 (34%)			
IV – *n* (%)			8 (25%)			
Ki67 index - median (range)	–		20 (2–70)		n.a.	
Weiss score - median (range)	–		7 (2–9)		n.a.	
**B. Cohort of patients for protein analysis by immunohistochemistry**							
		**ACA**		**ACC**		***p* value**	
*n* tissues		58		192			
*n* patients		58		166		*n.s*.	
Sex (F:M)		41:17		99:66		*n.s*.	
Age yrs-median (range)		52.5 (2–76)		46 (7–80)		*n.s*.	
Tumor size cm-median (range)		3.75 (1.5–14)		11 (3–30)		*< 0.05*	
Hormone secretion-*n* available		58		115		*n.s*.	
Cortisol – *n* (%)		20 (34.5%)		43 (37.5%)			
Androgen – *n* (%)		0 (0 %)		19 (16.5%)			
Aldosterone – *n* (%)		13 (22.4%)		5 (4.3%)			
Mixed – *n* (%)		0 (0%)		26 (22.6%)			
Inactive – *n* (%)		25 (43.1%)		22 (19.1%)			
Tumor localization		–				n.a.	
Primary tumor – *n*				147			
Local recurrences – *n*				25			
Metastases – *n*				20			
ENSAT Tumor Stage-*n* available		–		159		n.a.	
I–II – *n* (%)				80 (50.3%)			
III – *n* (%)				46 (28.9%)			
IV – *n* (%)				33 (20.8%)			
Ki67 index-median (range)		–		10 (1–80)		n.a.	
Weiss score-median (range)		–		5 (2–9)		n.a.	

Abbreviation: ACA: adrenocortical adenoma; ACC: adrenocortical carcinoma; n: number; F: female; M: male; yrs: years; n.a.: data not applicable; n.s.: p not significant.

The study was approved by the ethics committee of the University of Wuerzburg (No. 93/02 and 88/11) and written informed consent was obtained from all patients.

### Cancer cell lines

The livin/BIRC7 expression was also investigated in three different ACC cell lines: the reference NCI-H295 cell line and the adherent variant NCI-H295R (both steroid-secreting) as well as SW13, a non-steroid secreting cell line. NCI-H295 and NCI-H295R were cultured as previously described [56, 57]. SW13 were cultured in L15 Leibowitz medium with 10% FCS and 1% L-glutamine. STR profiling confirmation was performed on all the cell lines. Mycoplasma contamination PCR tests have been performed regularly and cells were maintained mycoplasma-free.

For the *livin* isoforms expression in ACC cell lines, two human neuroblastoma cell lines (IMR32 and SKN-MC) and Hela cells were used as positive controls, as previously reported [13, 22]. These three cell lines were cultured as previously described in DMEM-AQ medium with 10% FCS [57].

All cell lines were available in our laboratory and have been reauthenticated using STR-Analysis on February 2016 in the Department of Clinical Chemistry and Laboratory Medicine, University Hospital Würzburg.

### Quantitative real-time reverse transcription PCR

*Livin*, its isoforms *α* and *β*, *CASP3*, *XIAP* and *DIABLO* mRNA expression levels were analyzed by quantitative real-time PCR (qRT-PCR). In brief, RNA was isolated from fresh frozen tissue samples using the RNeasy Lipid Tissue Minikit (Qiagen, Hilden, Germany) and from cells using the RNeasy Mini Kit (Qiuagen). Reverse transcription of 1 μg of RNA was performed using the QuantiTect Reverse Transcription Kit (Qiagen) according to manufacturer's recommendations. Predesigned Taqman^®^ gene expression assays for *livin* (Hs01086675_m1), *CASP3* (Hs00234387_m1), *XIAP* (Hs00745222_s1) and *DIABLO* (Hs00219876_m1) (Applied Biosystems, Darmstadt, Germany) were used. For *livin α* and *β* isoforms we designed the amplification primer and the TaqMan hybridization probes spanning the 5′-part of exon 6 for *livin β* (Supplementary Table S2A). *Beta actin* (Hs9999903_m1) expression was used for normalization. For each PCR reaction 40 ng cDNA were used as template and each reaction was performed in duplicate. Amplification was performed using the TaqMan Gene Expression Master Mix (Applied Biosystems) and the CFX96 real-time thermocycler (Bio-Rad, California, USA). Cycling conditions were: 95°C for three min followed by 50 cycles of 95°C for 30 sec, 60°C for 30 sec, and 72°C for 30 sec. Transcript levels were determined using Bio-Rad CFX Manager 2.0 software and normalized to those of housekeeping gene using the ΔCT method (Pfaffl), as previously described [58].

### Reverse transcription-PCR

To compare the expression of the livin isoforms *α* and *β* in ACT and in adjacent NAG and in ACC cell lines, we additionally performed regular RT-PCR followed by size differentiation agarose electrophoresis. The primers used amplify a fragment including the 5′-part of exon 6, deleted in *livin β* [22] (Supplementary Table 2B). The expression of endogenous β2-microglobulin [18] was used for normalization. Primers were purchased from Eurofins Genomics (Ebersberg, Germany). RT-PCR was performed using the Sybr Green PCR Master Mix (Life Technologies, Warrington, UK). PCR products were identified by 4% agarose gel electrophoresis. We differentiated between two fragment sizes: 216 bp band for livin α and 162 bp for livin β. After gel documentation (Biometra, Göttingen, Germany) a semi-quantitative evaluation of the bands was performed using the Image Studio software 4.0 (LI-COR, Bad Homburg, Germany). Hela cells were used as positive control [13].

### Western blot analysis

For both WB and immunostaining analysis, we selected a rabbit polyclonal antibody (Ab) raised against livin (NB100-56145, Novus Biologicals, Hamburg, Germany, 1:1000), for which a synthetic peptide corresponding to amino acids 180–230 of human livin was used as immunogen (full-length recombinant human livin).

For the WB analysis, proteins were extracted from fresh-frozen tissues and cells as previously described [56, 59]. Tissues samples were homogenized by sonication at 4°C in RIPA buffer (Sigma-Aldrich, Missouri, USA) supplemented with Protease Inhibitor Cocktail (Sigma-Aldrich). Whole cell lysate SK-MEL 28 (sc-2236, Santa Cruz Biotechnology Inc., Heidelberg, Germany), derived from the melanoma CSK-MEL-28 cell line, was used as positive control for WB [60]. Electrophoresis of equal amounts of proteins was performed in a 12% SDS-polyacrylamide gel and transferred onto a PVDF membrane by tank-blotting. The membrane was blocked with 5% skim milk in TBS-Tween and incubated overnight at 4°C with the primary Ab against livin (dilution 1:1000). After washing, the membrane was incubated with horseradish peroxidase-labeled goat anti-rabbit IgG (NA934-100UL, Ge Healthcare, Little Chalfont, United Kingdom, 1:5000). The antigen-antibody complex was visualized by enhanced chemiluminescence using an Amerscham ECL reagent (WesternSure PREMIUM Chemiluminescent Substrate, LI-COR). Normalization of protein levels was performed by re-probing the blot with the antibody recognizing human β-actin (Sigma-Aldrich, 1:5000). A scan of the membrane was performed by C-DiGit Blot Scanner (LI-COR) and the quantification of individual bands was measured using the Image Studio software 4.0 (LI-COR). Two specific bands of 39 and 37 kDa corresponding to livin α and livin β, respectively, were detected in both positive control and adrenal tissues.

### Immunohistochemistry

Full serial sections and TMA were deparaffinized with xylol and rehydrated in descending graded series of ethanol. Immunohistochemical detection was performed with an indirect immunoperoxidase technique after high temperature antigen retrieval in 10 mM citric acid monohydrate buffer (pH 6.5) in a pressure cooker for 13 min. Blocking of unspecific protein–antibody interactions was performed with 20% human AB serum in PBS for 1 hour at room temperature. Tissue sections were incubated at room temperature for 1 hour with the primary polyclonal rabbit anti-livin Ab (NB100-56145, Novus Biologicals, 1:1000). As negative control, tissue sections were incubated with N-Universal Negative Control Anti-Rabbit (Dako, Glostrup, Denmark). Signal amplification was achieved at room temperature by En-Vision System Labeled Polymer-HRP Anti-Rabbit (Dako) for 40 min followed by 10 min development with DAB Substrate Kit (Vector Laboratories, California, USA) according to the manufacturer's instructions. Nuclei were counterstained with Mayer's hematoxylin for 2 min. As positive control, tissue sections from melanoma and renal clear cells carcinoma were stained.

All slides were analyzed independently by two investigators blinded to clinical information (B.A. and S.St.). Cytoplasmic livin staining was evaluated in all tissue samples measuring the staining intensity and the percentage of positive cells. The staining intensity was graded as negative (0), low (1), medium (2), or strong (3). The percentage of positive cells was scored as 0 if 0% were positive, as 0.1 if 1–9% were positive, as 0.5 if 10–49% were positive, and as 1 if 50% or more were positive. A semiquantitative H-score for the cytoplasmic localization was then calculated multiplying the staining intensity grading score by the proportion score as described previously [61]. Nuclear staining of livin was also evaluated in the group of adrenocortical tissues and the percentage of positive nuclei was scored as 1 if 0%, as 2 if 1–49%, and as 3 if 50% or more were stained. When discrepancies were observed, results were jointly assessed by both investigators and the final score was decided by consensus. Inter-observer agreement was strong, with a Spearman's correlation coefficient of 0.87 (95% CI 0.84–0.90).

All images were acquired by light microscopy (Biorevo BZ-9000, Keyence) using same light intensity parameters to avoid biased information. Original images were used for the analysis; printed images were modified in brightness and contrast.

### Relationship between livin and survivin immunostaining

We took advantage from the data already available in our institute about survivin/BIRC5 protein expression in a large series of adrenal samples [36]. Thus, we could investigate the relationship between livin and survivin cytoplasmatic immunostaining, and their potential synergistic role on clinical outcome, in a subgroup of 146 adrenal samples (118 ACC, 20 ACA and 8 NAG) where data were available for both proteins.

### *In vitro* experiments

NCI-H295R cells were cultured as previously described and grown as monolayers in DMEM/F12 (1:1) medium (Gibco, Invitrogen Ltd., UK) supplemented with 5 ml of insulin, transferrin and selenium solution (IST, Gibco, 41400-045) and 2.5% Nu-serum (Corning, Thermo Fisher Scientific, Manasses, VA, USA) at 37°C and 5% CO_2_ [56, 57]. One day prior to transfection, 6 × 10^5 cells were seeded in 6 well plates. 2 μg of vector DNA were diluted in 200 μl OPTI-MEM serum-free media (Gibco) and 6 μl of the X-treme GENE HP DNA transfection reagent (Sigma Aldrich) was added and incubated 20 min at room temperature. Transient *livin* overexpression was induced in the ACC cell line NCI-H295R by using a TrueORF Gold Myc-DDK-tagged livin cDNA clone (RC204906, OriGene, Herford, Germany). An empty pCMV6-Entry vector was used as control. The *livin* transfection rate was confirmed by qRT-PCR and WB and all experiments were conducted at least in triplicates. As a marker of apoptosis, we evaluated the *CASP3* expression by qRT-PCR and the cleaved caspase-3 by both WB and IF. qRT-PCR for *livin α*, *livin β* and *CASP 3* was performed as earlier described. For WB analysis, the protein lysates were blotted onto nitrocellulose membranes and incubated with livin antibody (1:1000), cleaved caspase-3 antibody (#9661, Cell Signaling, 1:250) and α-tubulin antibody for the normalization of protein levels (clone DM1A, T9026, Sigma-Aldrich, 1:40000). Because we observed significant results after 72 hours, we decide to proceed with IF at this time point. For IF, cells were grown in chamberslides and transfected as described. After 72 h hours, cells were subsequently fixed in 4% paraformaldehyde in PBS for 8 min, washed with PBS and permealised with 0.5% Triton X100 in PBS for five minutes. After washing, cell were incubated overnight at 4°C with mouse anti-FLAG antibody (clone M2, F1804, Sigma-Aldrich, 1:50) and rabbit anti-cleaved caspase 3 antibody (1:50). For detection, cells were incubated with Alexa Fluor-488 or -594 (Molecular Probes, Invitrogen) labeled anti-mouse or anti-rabbit secondary antibodies, respectively, at room temperature for one hour. Cells were mounted with Vectashield antifade mounting medium containing DAPI (H-1200, Vector Laboratories, Burlingame, CA, USA). Image acquisition was performed using a flourescence microscope (Axiovert 135, Carl Zeiss). Merged images were obtained using the corresponding software (AxioVision, Carl Zeiss). Cell from three separate experiments were counted automatically with ImageJ software, for a total count of more than 1000 cells.

A cell viability test using the WST-1 proliferation reagent was performed according to the manufacturer's protocol (Roche Diagnostics Deutschland GmbH, Mannheim, Germany) at both 48 and 72 hours after the transfection. After adding WST-1 reagent, absorbance was measured using a microplate reader (1420 VICTOR3, PerkinElmer Inc., Waltham, MA, USA) after two hours. At least six wells were used for each experimental condition and all experiments were conducted in triplicates.

### Statistical analysis

The Fisher's exact test or the Chi-square test was used to investigate dichotomic variables, while a two-sided *t* test (or non-parametric test) was used to test continuous variables as appropriate. A non-parametric Kruskal-Wallis test, followed by Bonferroni *post-hoc* test, was used for comparison among several groups for non-normal distributed variables. Correlations and 95% confidence intervals (95%CI) between different parameters were evaluated by linear regression analysis. Overall survival (OS) was defined as the time from the date of primary surgery to disease-related death or last follow-up. Progression-free survival (PFS) was defined as the time from the date of complete tumor resection to the first radiological evidence of disease relapse or disease-related death. All survival curves were obtained by Kaplan-Meier estimates and the differences between survival curves were assessed by the log-rank (Mantel-Cox) test. In this context, the RNA expression was considered both as a categorical value (cut-off for this data set: median +2SD) and as an ordinal variable (high, medium and low expression: < 25%, 25–75%, and > 75% percentile, respectively). A multivariate regression analysis was performed by Cox proportional hazard regression model to identify those factors that might independently influence survival. Statistical analyses were made using GraphPad Prism (version 5.0, La Jolla, CA, USA) and SPSS Software (PASW Version 21.0, SPSS Inc., Chicago, IL, USA). *P* values *< 0.05* were considered as statistically significant.

## SUPPLEMENTARY TABLES AND FIGURES


