# Supplementary material for: Livin/BIRC7 expression as malignancy marker in adrenocortical tumors
Source: Oncotarget. 2016 Dec 21;8(6):9323–38. doi: 10.18632/oncotarget.14067 (PMC5354734; doi:10.18632/oncotarget.14067)
Supplement: Supplementary file 1 [file oncotarget-08-9323-s001.pdf]

Livin/BIRC7 expression as malignancy marker in adrenocortical tumors

Supplementary Materials

Supplementary Table S1: Correlation of progression-free and overall survival with mRNA expression of investigated genes

A. Progression-free survival			
**genes**	**Median survival-months**	**HR (95% CI of ratio)**	***p* value**
BIRC7			
high (n = 14)	23	0.75 (0.34–1.70)	*n.s.*
low (n = 15)	14	1.32 (0.58–1.96)	
Livin α			
high (n = 14)	23	0.69 (0.28–1.69)	*n.s.*
low (n = 15)	10	1.45 (0.59–3.57)	
Livin β			
high (n = 14)	23	0.67 (0.27–1.65)	*n.s.*
low (n = 15)	10	1.49 (0.61–3.66)	
CASP3			
high (n = 12)	5	1.42 (0.58–3.48)	*n.s.*
low (n = 17)	18	0.70 (0.29–1-71)	
BIRC4			
high (n = 14)	13.5	1.04 (0.42–2.53)	*n.s.*
low (n = 15)	19	0.9 (0.39–2.35)	
DIABLO			
high (n = 14)	21	0.58 (0.24–1.43)	*n.s.*
low (n = 15)	14	1.71 (0.69–4.16)	

B. Overall survival			
**genes**	**Median survival-months**	**HR (95% CI of ratio)**	***p* value**
BIRC7			
high (n = 14)	38	1.78 (0.59–5.31)	*n.s.*
low (n = 15)	48	0.56 (0.18–1.67)	
Livin α			
high (n = 14)	48	1.41 (0.47–4.23)	*n.s.*
low (n = 15)	undefined	0.71 (0.24–2.12)	
Livin β			
high (n = 14)	48	1.05 (0.35–3.15)	*n.s.*
low (n = 15)	36	0.95 (0.32–2.84)	
CASP3			
high (n = 12)	42	1.78 (0.59–5.34)	*n.s.*
low (n = 17)	undefined	0.56 (0.18–1.69)	
BIRC4			
high (n = 14)	36	0.97 (0.31–3.04)	*n.s.*
low (n = 15)	48	1.03 (0.33–3.20)	
DIABLO			
high (n = 14)	48	1.21 (0.38–3.81)	*n.s.*
low (n = 14)	100	0.82 (0.26–2.59)	

Abbreviation: n.s.: p not significant; HR: hazard ratio; CI: confidence interval.
RNA expression of all evaluated genes considered as a categorical value (cut-off for this data set: median +2SD). Statistical analysis by Kaplan-Meier survival curves and log-rank (Mantel-Cox) test in a total of 29 patients of which we had tissue from primary tumor. Hazard ratio by Mantel-Haenszel method.

Supplementary Table S2: Primers and probes used to investigate specific livin isoforms α and β

A. TaqMan amplification	Sequences
Forward amplification primer	5′ CCTCCGTCCCTGCCTCTG 3′
Reverse amplification primer	5′ TCCAGGCACACCTTGCAC 3′
TaqMan hybridization probe livin α	5′ TCAGTCCAGCCGAGGCCCAG 3′
TaqMan hybridization probe livin β	5′ AGGAGCCAGGAGCCAGGGAT 3′
**B. Size differentiation PCR***	**Sequences**
Livin	
forward	5′ ACCCAGGAGAGAGGTCCAGT 3′
reverse	5′ CACTCAGCACAGACCAGGTG 3′
β2-microglobulin control	
forward	5′ TTAGCTGTGCTCGCGCTACTCTCTC 3′
reverse	5′ GTCGGATTGATGAAACCCAGACACA 3′

*PCR primer sequences used for livin were taken from from Kim DK et al. [22] and those for β2-microglobulin from Gazzaniga et al. [18].

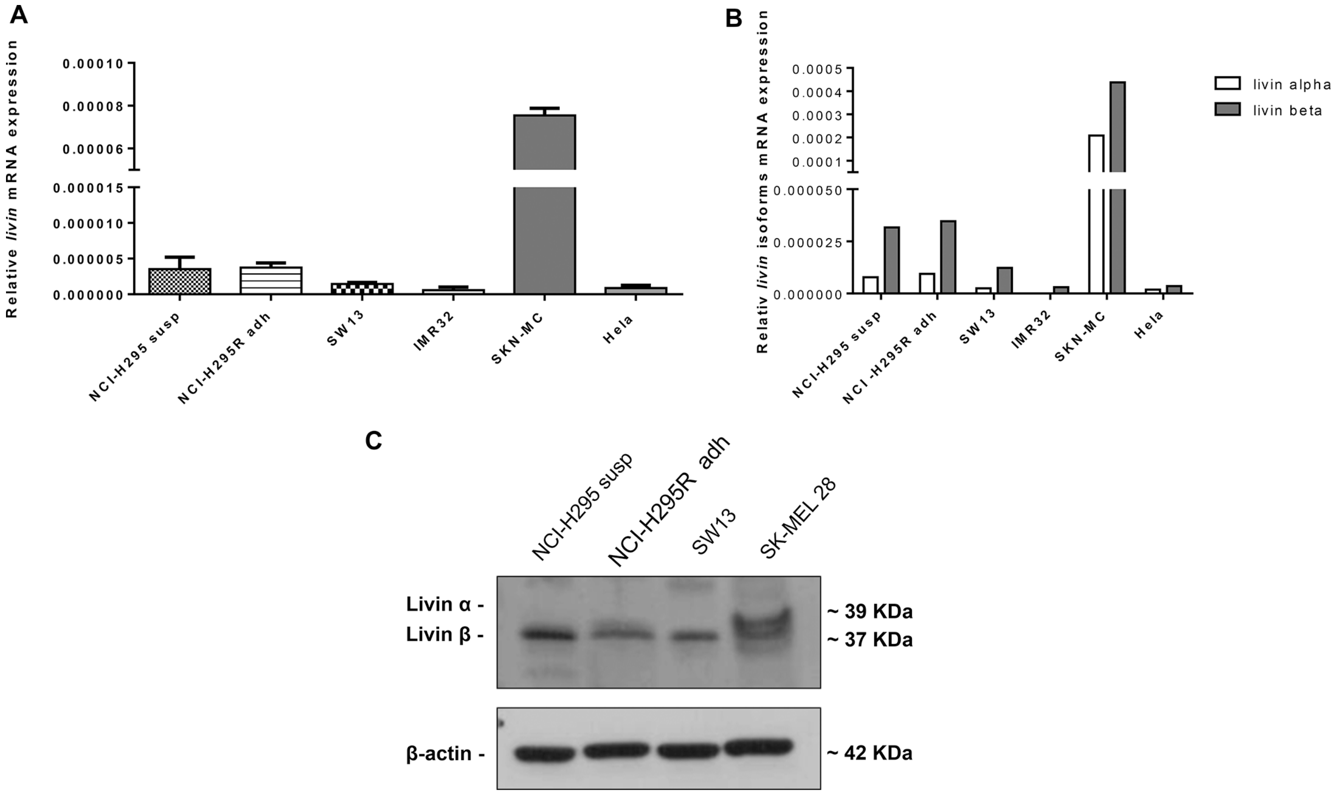

Supplementary Figure S1: Relative total *livin* and its isoforms α and β at mRNA and protein levels in ACC cell lines. (**A**–**B**) Relative total *livin* and *livin* isoforms *α* and *β* mRNA expression levels (by qRT-PCR) in three adrenocortical cancer cell lines (NCI-H295 (suspension), NCI-H295R (adherent) and SW13) in comparison with three controls, including two human neuroblastoma cell lines (IMR32 and SKN-MC) and HeLa cells. (**C**) Western blot analysis of livin isoforms expression (anti-livin antibody from Novus Bio, dilution 1:250) in NCI-H295 suspension, NCI-H295 adherent and SW13. Whole cell lysate SK-MEL 28 was used as positive control and β-actin was used as internal standard.

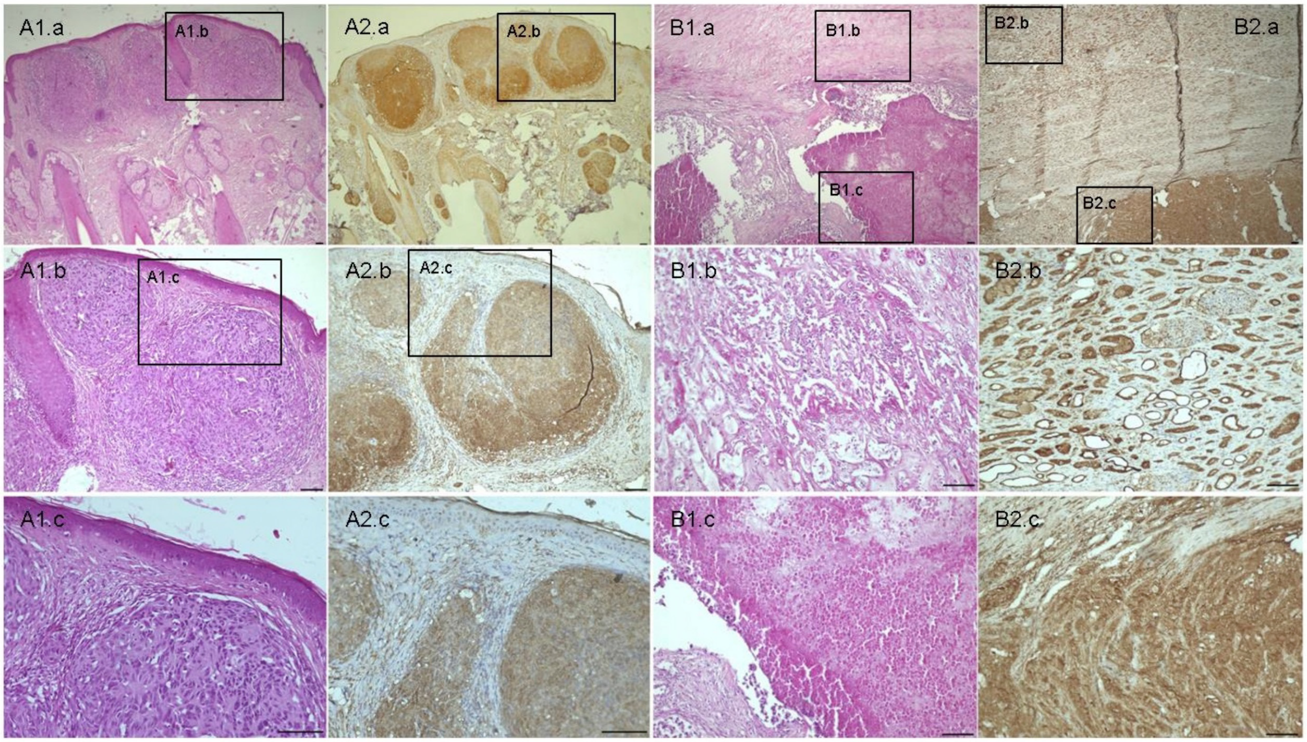

Supplementary Figure S2: Hematoxylin & eosin and livin staining on melanoma and renal cell carcinoma with corresponding adjacent normal tissues. (**A**) Skin melanoma with adjacent normal skin stained for H&E (A.1a–c) and livin (A.2a–c). The Figures A.1b, 2b and A.1c, 2c are 10× and 20× enlarged and detailed images of the 2× A.1a and A.2a, respectively. (**B**) Clear renal cell carcinoma with adjacent normal kidney stained for H&E (B.1a–c) and livin (B.2a–c). The figures B.1b, 2b and B.1c, 2c are 10× and 20× enlarged and detailed images of the 2× B.1a and B.2a, respectively. Scale bar: 100 µm.

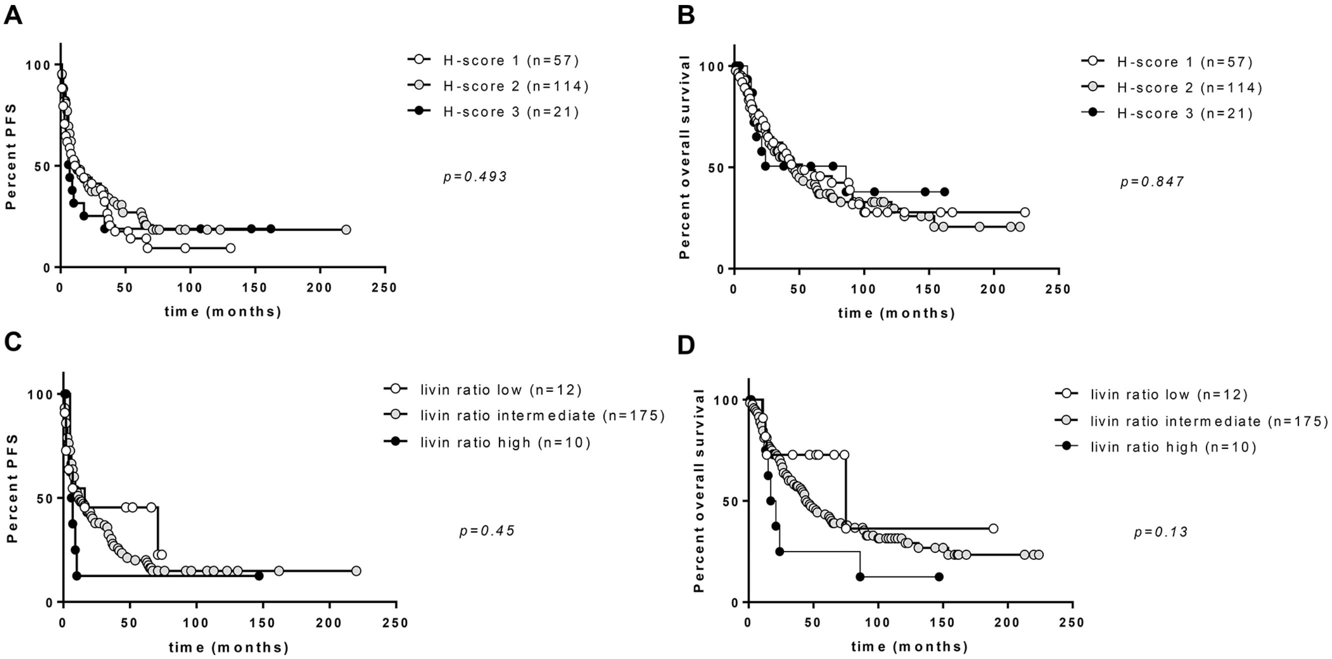

Supplementary Figure S3: Progression-free and overall survival for livin cytoplasmic protein expression and cytoplasmic/nuclear ratio. Progression-free (PFS) and overall survival analysis in 147 primary adrenocortical carcinomas for (**A**, **B**) cytoplasmic livin expression and (**C**, **D**) livin cytoplasmic/nuclear ratio. No significant impact on PFS and overall survival was found for both cytoplasm expression and cytoplasmic/nuclear ratio. Statistical analysis by Kaplan-Meier survival curves and log-rank (Mantel-Cox) test.

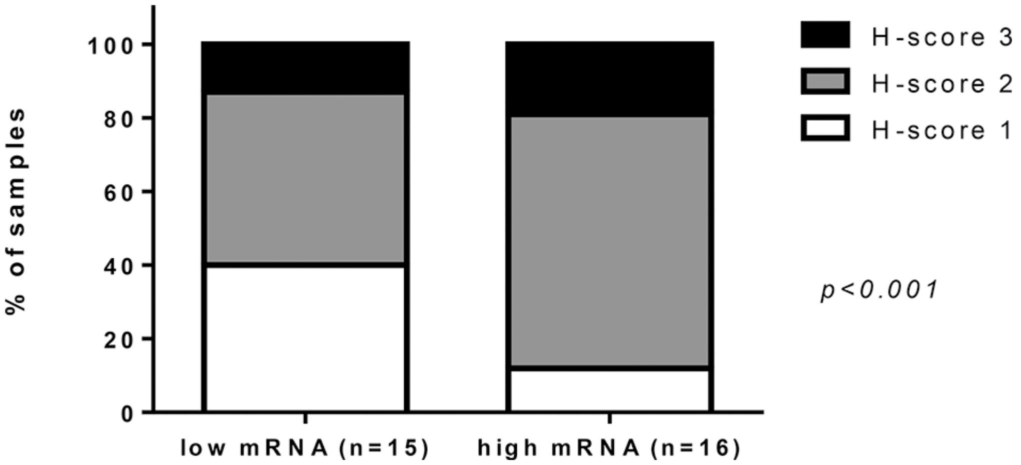

Supplementary Figure S4: Relationship between livin cytoplasmic staining and *livin* mRNA level. Relationship between livin cytoplasmic protein expression evaluated as H-score and the relative *livin* mRNA level expressed as low or high of the median ΔCT values in 31 adrenocortical tumors (10 adenomas, among which 9 expressed low *BIRC7* mRNA level, and 21 carcinomas, among which 15 presented expressed high *BIRC7* mRNA level). Statistical analysis by Chi-squared test.

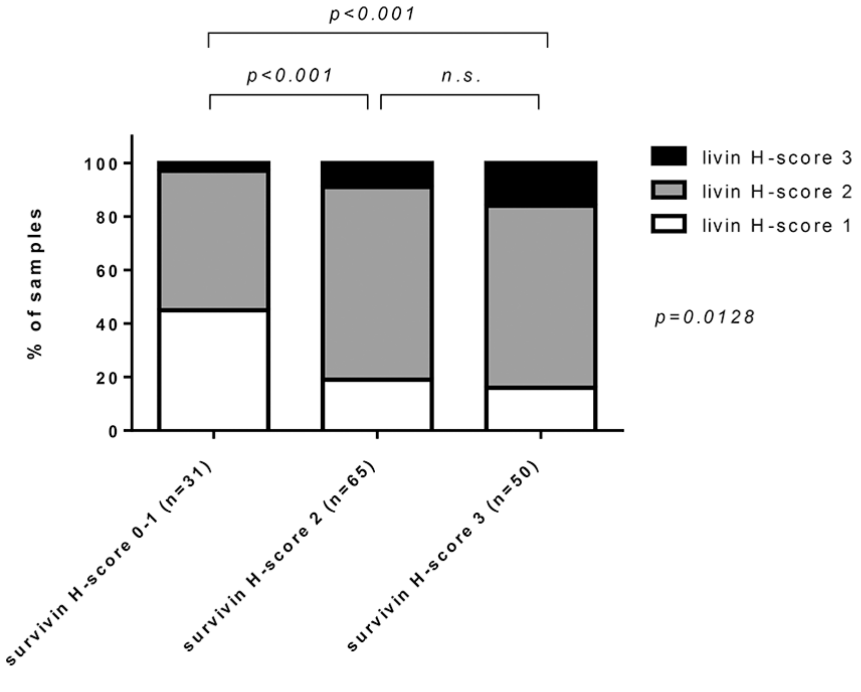

Supplementary Figure S5: Relationship between livin and survivin. Relationship between livin and survivin protein expression evaluated by H-score in a subgroup of 146 adrenal samples. The data about survivin protein expression are taken from Sbiera et al. 2013 [36].

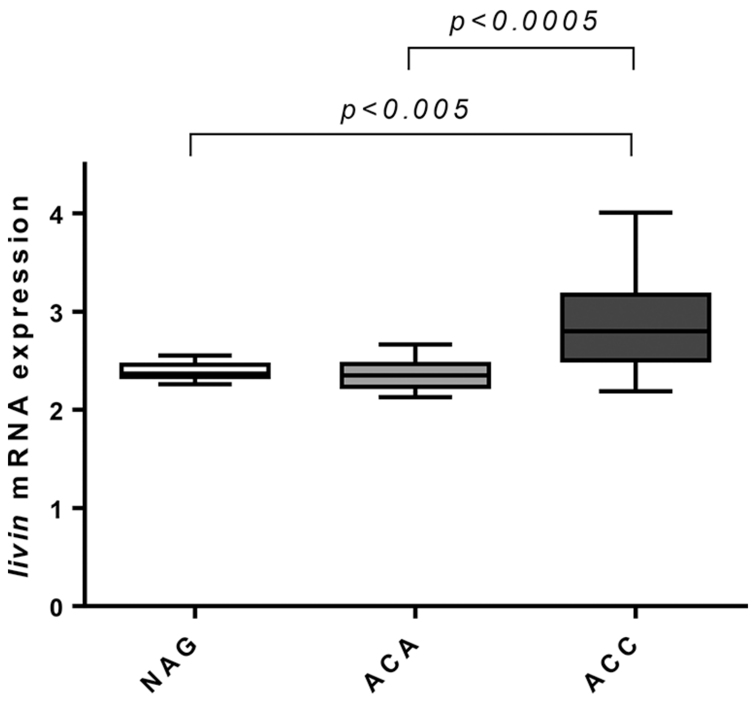

Supplementary Figure S6: *In silico* analysis of *livin* mRNA expression levels. *Livin* mRNA expression was extracted from the gene expression array data on 10 normal adrenal glands (NAG), 22 adrenocortical adenomas (ACA) and 33 adrenocortical carcinomas (ACC) published by Giordano et al. [38]. Statistical analysis by Kruskall-Wallis test.
